# Discussion of the Fetus in Fetal Cardiology Consultations: A Qualitative Study

**DOI:** 10.3390/children12091211

**Published:** 2025-09-10

**Authors:** Samantha Syme, Kelsey Schweiberger, Judy C. Chang, Ann Kavanaugh-McHugh, Abdesalam Soudi, Justin T. Clapp, Nadine A. Kasparian, Robert M. Arnold, Kelly W. Harris

**Affiliations:** 1 University of Pittsburgh School of Medicine, 3550 Terrace Street, Pittsburgh, PA 15213, USA; 2 Department of Pediatrics, University of Pittsburgh School of Medicine, 4221 Penn Avenue, Pittsburgh, PA 15224, USA; kelsey.schweiberger@chp.edu (K.S.); harriskw@upmc.edu (K.W.H.); 3 Department of Obstetrics, Gynecology, and Reproductive Sciences, University of Pittsburgh School of Medicine, 300 Halket Street, Pittsburgh, PA 15213, USA; chanjc@upmc.edu; 4 Department of Pediatrics, Vanderbilt University Medical Center, 2200 Children’s Way, Suite 2404, Nashville, TN 37232, USA; ann.kavanaugh-mchugh@vumc.org; 5 Department of Linguistics, University of Pittsburgh, 4200 Fifth Avenue, Pittsburgh, PA 15260, USA; soudia@pitt.edu; 6 Department of Medical Ethics & Health Policy, University of Pennsylvania Perelman School of Medicine, 23 Guardian Drive, Blockley Hall, 11th & 14th Floors, Philadelphia, PA 19104, USA; justin.clapp@pennmedicine.upenn.edu; 7 Heart and Mind Wellbeing Center, Heart Institute and Division of Behavioral Medicine and Clinical Psychology, Cincinnati Children’s Hospital Medical Center and Department of Pediatrics, University of Cincinnati College of Medicine, 3333 Burnet Avenue, Cincinnati, OH 45229, USA; nadine.kasparian@cchmc.org; 8 Department of Geriatrics and Palliative Medicine, Icahn School of Medicine at Mount Sinai, 1468 Madison Avenue, New York, NY 10029, USA; robert.arnold@mssm.edu

**Keywords:** fetal cardiology, terminology, CHD, prenatal diagnosis, qualitative research

## Abstract

**Highlights:**

**What are the main findings?**

- Fetal cardiology clinicians primarily used personal terminology when referring to the fetus in initial fetal cardiology consultations.
- Clinicians often attributed agency and/or mental states to the fetus.

**What is the implication of the main finding?**

- This is the first study to define and explore the use of agency and mental states in prenatal counseling appointments; the effects of this language need to be explored further.
- Understanding what terminology is used in initial fetal cardiology consultations is an imperative step in optimizing communication in this setting.

**Abstract:**

**Background:** While prenatal diagnosis of congenital heart disease is increasingly common, and communication is essential to minimizing familial stress, little is known about how the fetus is discussed in this setting. This study observed how clinicians and families refer to the fetus during initial fetal cardiology consultations. **Methods:** Initial fetal cardiology consultations from one institution were recorded and transcribed verbatim. A codebook was developed and used to code the transcripts. Codes included any reference to the fetus and any attribution of agency or mental states to the fetus. **Results:** Nineteen consultations performed by five clinicians from one academic institution were included. Clinicians and families most frequently referred to the fetus using personal terminology (e.g., third-person pronouns, a given name, or “son” or “daughter”). Impersonal terminology (e.g., “baby”) was used less frequently, followed by medical terminology (e.g., “fetus”), which was only used in two consultations. In about half of the consultations, clinicians conferred agency or mental states on the fetus by attributing actions, emotions, or knowledge to the fetus. **Conclusions:** Fetal cardiology clinicians primarily use personal terminology when referring to the fetus during initial consultations. Familial preferences need to be evaluated to optimize communication and support.

## 1. Introduction

Each year, 1.3 million children worldwide are born with congenital heart disease (CHD) [1]. The prenatal detection of complex CHD, defined here as Grade 3 or higher on the Fetal Cardiovascular Disease Severity Scale [2], has increased with routine fetal echocardiography—83% of complex CHD diagnoses are now made prenatally [3]. In this context, effective communication is important as it can greatly affect parents’ psychological adjustment, which can further impact children’s developmental outcomes [4,5,6]. However, a recent survey of pediatric cardiologists who provide counseling for prenatal CHD found that less than half of providers had received communication training in this area [7].

Despite increased prenatal detection, little is known about how fetal cardiologists refer to the fetus in the setting of prenatal complex CHD diagnosis, and there is no consensus-based framework for how to refer to a fetus when discussing complex CHD prenatally. (The term “fetus” is used throughout this manuscript as medical terminology in place of an agreed-upon word.) In other prenatal settings with high fetal mortality risk or where abnormal sonography findings are discussed, families prefer the term “baby” rather than “fetus” [8,9]. Similarly, in cases of extreme prematurity, families report that terms such as “baby”, “son or daughter”, “child”, or “[given name]” are preferred [10]. While we assume similar terminology is used in consultations for prenatal diagnosis of complex CHD, this topic has never been formally studied before and is an essential step in assessing how to improve communication training in this specific setting. This study aimed to identify current clinician and familial practices regarding how the fetus is referred to during initial fetal cardiology consultations following prenatal complex CHD diagnosis.

## 2. Methods

### 2.1. Study Design and Data Collection

This paper presents a secondary qualitative analysis of verbatim transcriptions of audio recordings of initial fetal cardiology consultations collected as part of a larger study [11,12]. Prior analyses focused on the discussion of uncertainty and discourse styles used in these conversations [11,12]. The current study analyzed a different set of newly developed and applied codes, focused specifically on terminology referring to the fetus, which has not been explored in prior analyses or publications. The methods used in this analysis are further delineated below.

Clinicians were recruited via one tertiary care facility; all clinicians who conducted fetal cardiology consultations at the hospital were invited to participate in the study via email. The study was then discussed in detail with all interested clinicians prior to enrollment.

Pregnant and accompanying individuals at the consultation were eligible if they had been referred for a possible complex CHD diagnosis and were presenting for their initial fetal cardiology consultation. Non-native English speakers were included in the study if an in-person interpreter was present or if they chose to conduct the consultation in English. Pregnant individuals were approached in person regarding interest in the study immediately prior to the consultation.

All individuals present for the initial fetal cardiology consultation provided written and verbal consent to participate in the study prior to the consultation. Participants included clinicians, as well as pregnant and accompanying persons (hereafter referred to as families). The counseling component of the consultations was audio-recorded using a digital voice recorder placed in the counseling room; any verbal exchange that occurred during the fetal echocardiogram in a different physical space was not audio-recorded.

Demographic information was collected by study personnel prior to the consultation. All aspects of the study were approved by the appropriate Institutional Review Board.

### 2.2. Data Analysis

Audio recordings were transcribed verbatim, and all identifying information was removed. For consultations using an interpreter, the interpreter’s statements were transcribed in English, and the original communication was not transcribed. Content analysis was carried out using both inductive and deductive approaches. Using a deductive approach, terminology used to refer to the fetus was grouped into distinct codes using linguistic expertise. Specific deductive codes were developed based on the linguistic literature [8,9,13,14,15] to apply uniquely to this clinical situation, capturing references to relational and agentive identities of the fetus. Recognizing the lack of empirical research into how fetal cardiology clinicians refer to the fetus during discussions with families, we also used inductive codes to capture patterns identified during consultations [16]. Inductive codes included codes for mirroring, agency, and mental states. These were all nuances that were noticed throughout the consultations, with codes then developed to capture their use.

A preliminary codebook was created by two coders (S.S. and K.W.H.) to code word use (e.g., any reference to the fetus, mirroring terminology), including definitions, inclusion and exclusion criteria, and examples. The codebook was then iteratively refined by S.S. and K.W.H., with input from K.S. and J.C. Once consensus was reached, the final codebook was applied to all consultations by S.S.; K.W.H. independently coded 20% of the consultations (*n* = 4) to ensure intercoder reliability; agreement was >90% [17]. The NVivo14 software (QSR International; Burlington, MA, USA) was used to store and organize data.

In our analysis, we coded single words or phrases that referred to the fetus at any point during the recorded conversation, spoken by clinicians or by families. We then examined patterns and relationships between codes over time during the consultation. The frequency of clinician and family use of each code per consultation was tabulated for context.

## 3. Results

### 3.1. Demographics

Of the seven clinicians invited to take part in the study, five participated, one declined, and one had no eligible patients during the study period. Clinicians had received medical training across five institutions and had been practicing at the study institution for a median of 10 years (interquartile range of 2 years) at the time of the study. Of the 31 pregnant individuals invited to participate, 19 participated, 5 declined, and 7 were ultimately ineligible based on normal fetal echocardiograms during the consultation. Most pregnant individuals identified as white and non-Hispanic (14; 74%) and reported English as their primary language (15; 79%). Median gestational age at the time of consultation was 26 weeks, and about half (9; 47%) of the consultations pertained to a diagnosis with a higher mortality risk, defined as a score ≥6 on Allan’s (2004) scale [18]. Most pregnant participants (14; 74%) were accompanied by another person (i.e., partner, parent, or friend of the pregnant individual). The full demographic information has been published previously [11].

### 3.2. Categories of Terminology Used by Families and by Clinicians

Terminology used by families and by clinicians when referencing the fetus fell into three main categories: medical, impersonal, and personal (Table 1). Medical terminology referred to any use of the word “fetus”. Impersonal terminology was defined as words that do not have a human connotation or imply a relationship, such as the word “baby”. Personal terminology included any use of pronouns, “son/daughter”, or a given name if provided by the family. Additionally, some providers mirrored the terminology used by families. Mirroring was identified if the clinician used the same terminology as the family after the family first mentioned the fetus or if the clinician used the given name noted on the clinic intake forms. In addition to using specific terminology, families and clinicians referenced the fetus by attributing agency or mental states to the fetus. We identified agency when actions were attributed to the fetus and mental states when emotions and/or knowledge were.

### 3.3. Terminology Used by Families

On average, families spoke 11% of the words uttered in the consultation (range: 2% to 23%). In total, 15 of the 19 families (79%) referred to the fetus; personal terminology was used most frequently, and medical terminology was used least frequently (Table 2). In total, 10 of the 19 pregnant individuals (53%) reported having decided on a name for the fetus. There was only one instance of a family attributing agency to the fetus; in that case, the father described the fetus’s vigorous movements as “playing soccer” (Family 14).

### 3.4. Language Used by Clinicians

#### 3.4.1. Mirroring

In 8 of the 19 consultations (42%), the fetal cardiologist mirrored the terminology families used after the first mention of the fetus. For example, in one consultation, a family asked, “So, **he** does have a hole [in his heart]?” and the clinician mirrored the family’s personal terminology by responding, “He does have this” (Family 17).

#### 3.4.2. Terminology Used by Clinicians

Clinicians used a variety of terms to refer to the fetus, ranging from medical terminology such as “fetus” to personal terminology such as the given name (Table 1). Clinicians rarely used the word “fetus” or any variation of this word; “fetus” was used twice across all 19 consultations. Personal terminology was used most frequently; personal third-person pronouns (e.g., she or he), a given name, and “girl” or “boy” were used in every consultation. There was an average of 78 uses of personal terminology by clinicians per consultation, out of an average of 82 total references to the fetus by clinicians per consultation. Terminology use did not seem to differ by the mortality risk associated with the cardiac diagnosis.

### 3.5. Personal Terminology: Agency and Mental State Attribution

Within clinician use of personal terminology, terminology attributing agency or mental states to the fetus was used in 10 of 19 (53%) consultations. Fetal cardiology clinicians attributed agency by describing actions of the fetus (eight consultations, two clinicians; Table 2). Terminology chosen by clinicians attributed mental states by referencing the fetus’s emotions (four consultations, three clinicians) or knowledge (nine consultations, two clinicians).

Attribution of agency or mental states did not seem to differ based on sharing of a given name or mortality risk associated with a complex CHD diagnosis. For example, when discussing a fetus with a higher risk of mortality, the clinician attributed agency by stating, “He’s an active little guy… you can feel him living” (Family 7). When discussing a fetus with a lower risk of mortality, another clinician said, “She doesn’t know that she’s any different right now, okay? She’s rolling around” (Family 8). These examples highlight the use of agency and mental states, regardless of prognosis.

## 4. Discussion

In this study, when discussing a new prenatal diagnosis of complex CHD, both fetal cardiology clinicians and families most frequently referred to the fetus using personal terminology. Impersonal and medical terminology were used less frequently. In over half of the consultations, the terminology chosen attributed mental states or agency to the fetus by referencing emotions or knowledge, or by describing actions. This study significantly adds to the literature by expanding our knowledge of how fetal cardiology clinicians communicate with families and specifically exploring, for the first time, how mental states and agency are used in the setting of a prenatal diagnosis.

Clinicians’ use of personal terminology is consistent with previously identified family preferences for non-medical terminology when referring to a fetus in other settings (e.g., extreme prematurity or abnormal sonogram) [8,9,10,11]. In this study, we also further delineated terminology categories used, breaking down non-medical terminology into personal and impersonal. Family preferences among these categories need to be explored further. Personal terminology inherently gives the fetus “animacy” and relational ties to the family, thus personalizing what is likely a difficult conversation for all involved [19,20]. In other prenatal contexts, clinicians reported using personalization to connect with families [13,19]. While family preferences around terminology were not explicitly elicited in this study, the use of mirroring by clinicians suggests an attempt to match families’ chosen terminology. Prior research has suggested that clinicians use mirroring to reflect a respect for and acknowledgement of families’ preferences and bond with their fetus [21,22]. Additionally, in the linguistic literature, repetition of words in the context of conversation can demonstrate engagement [23]. While the existing literature describes why clinicians use mirroring, we describe, for the first time, how this mirroring occurs. Family views on mirroring need to be explored further. In our study, the clinicians’ use of terminology did not seem to differ based on the risk of mortality associated with a complex CHD diagnosis, suggesting prognosis did not affect clinicians’ terminology when referring to the fetus.

Attribution of agency and mental states by clinicians added further personalization to their counseling. In more than half the consultations, the fetus was described as carrying out actions or having knowledge or emotions. We know anecdotally that attribution of these qualities to the fetus is used throughout prenatal care, but this is the first study, to our knowledge, to look at clinicians’ use of agency and mental states explicitly when providing medical counseling. There have been a few ethnographic studies looking at the use of agency by tangential clinicians, such as medical imaging technicians. One study found that sonographers, for example, may personalize a fetus by attributing emotions or intention; for example, they may say “he keeps hiding” when having difficulty obtaining a particular view of the fetus [24]. This demonstrates that there may be similarities between how sonographers and fetal cardiologists use agency when talking to families. However, to our knowledge, this is the first study to incorporate linguistics to explicitly name these concepts—agency and mental states—and describe their use in the context of prenatal diagnosis. Based on the authors’ clinical expertise, sometimes clinicians may attribute agency or mental states to translate complex medical jargon into terminology that is more broadly understandable. The purpose and potential effects of personal terminology use, including attribution of agency and mental states, during prenatal consultations should be explored further.

### 4.1. Implications and Next Steps

This study highlights the consistent use of personal terminology and intermittent use of mirroring during initial fetal cardiology consultations following prenatal complex CHD diagnosis. Future studies should consider all methods of communication (e.g., electronic messages or short verbal exchanges outside the formal counseling session) and assess both clinician intentions and family preferences regarding terminology strategies. Understanding whether families would like to be asked directly about terminology preferences and, if not, which terminology should be assumed is imperative. If they do prefer to be asked about their preferences, it is necessary to learn how best to ask and how to re-evaluate preferences as the pregnancy progresses to help shape best practice guidelines. We hypothesize that parents may, for example, prefer for the given name and pronouns for their fetus to be asked at every appointment, similar to what is recommended for assessing preferred name and pronouns at adolescent patient appointments [25].

Various terminology choices could have significant implications for parent and family wellbeing and decision-making. In the United States, in the context of contemporary debates and changing state laws around pregnancy termination following the overturning of *Roe v. Wade*, clinician word choice for discussing a fetus could affect parental perceptions in profound and potentially unintended ways. In June 2022, after data collection for this study had been completed, *Roe v. Wade*, a Supreme Court case that protected the right to pregnancy termination before the point of viability nationwide, was overturned [26]. In the United States, termination regulations are now determined by each state’s individual constitution. At this time, the state where this study was conducted does not allow termination except in medical emergencies. It is, therefore, essential to further understand family preferences in the larger context of laws regarding termination and how the changing landscape may affect these preferences. After investigating these areas, findings should be applied to the development of communication training for clinicians to optimize clinician–family communication in the context of a prenatal complex CHD diagnosis.

### 4.2. Strengths and Limitations

This is one of only a few studies to examine the terminology used to refer to the fetus during a prenatal visit. This study is unique in directly analyzing terminology in fetal cardiology consultations and via audio recordings transcribed verbatim, as opposed to asking participants to recall the terminology utilized [21]. It is also the first time the use of agency and mental states has been explicitly described and studied in the context of prenatal diagnosis of serious illness.

Despite these strengths, the findings of this study should be interpreted in the context of a few limitations. All the participating clinicians were recruited from one academic institution, and their practices may not be reflective of the practices at other institutions. Family participants predominantly specified English as their native language (79%); only one consultation used an interpreter. Additionally, all participating families had access to a fetal cardiology clinic, potentially limiting generalizability. Most of our analysis focused on the clinicians’ terminology due to the paucity of words spoken by families. While consultations were recorded and transcribed verbatim, review of intake forms and introductory discussions may have occurred outside of the recording. Thus, we may have missed introductions to the terminology used or stated preferences by the family.

Given the median gestational age in our study was 26 weeks, terminology used by clinicians and families may differ at an earlier gestational age, such as before fetal viability. Previous research has demonstrated that family preferences, which include terminology, may not be stagnant throughout pregnancy [15]. Therefore, it is important to note that terminology use and preferences may be fluid, which was not explored in this study.

## 5. Conclusions

Fetal cardiologists in this study most frequently used non-medical, personal terminology when referring to the fetus in conversations with families about a new prenatal diagnosis of serious illness. Future studies should investigate family and clinician terminology preferences and potential benefits and consequences associated with the use of personal terms, mirroring, and agency and mental states in clinician–family communication following prenatal diagnosis of congenital heart disease.

## Figures and Tables

**Table 1:** Types of language used to refer to and discuss the fetus.

Use of Language		Definition		Examples/Quotes
**Mirroring**		Clinician matches family’s terminology after their first mention of the fetus or uses the given name written on intake forms		Father: “**She** won’t feel it?” Cardiologist: “**She** won’t notice that **she’s** blue, no”. Family 18
**Medical**		Words refer to stage of development		Mother: “We thought we were having little **fetal movements**”. Family 14
**Impersonal**		Does not imply personhood or a relationship		Cardiologist: “Every little bit that you do is going to be helpful for the development and growth of **this baby**”. Family 17
**Personal**	**Word Choice**	Words that have a human connotation, including pronouns, chosen name		Cardiologist: “I’m going to go over what a normal heart looks like, how **your son’s** heart is different, and then what we’re going to do about it”. Family 11
	**Agency or** **Mental States**	**Actions**	Describes the fetus “doing” something	Cardiologist: “But at this point, that’s where we are and we just have to watch to see what your little guy is going to **show** us”. Family 5
		**Emotions**	References feelings of the fetus	Cardiologist: “He is completely **happy**”. Family 11
		**Knowledge**	Notes that the fetus has awareness	Cardiologist: “But right now, as I said, she **does not know** she is different than anyone else”. Family 7

**Table 2:** Frequency of language used by families and clinicians to refer to and discuss the fetus.

Use of Language			Frequency of Use by Family ^a^	Frequency of Use by Clinician ^b^
**Mirroring**			N/A	8 consultations
**Medical**			1 consultation	2 consultations
**Impersonal**			5 consultations	All consultations; average of 10 uses per consultation
**Personal**	**Word Choice**		All consultations where the fetus was referenced (15/19); average of 6 uses per consultation	All consultations; average of 78 uses per consultation
	**Agency or** **Mental States**	**Actions**	1 consultation	7 consultations ^c^
		**Emotions**	Never used	4 consultations ^c^
		**Knowledge**	Never used	9 consultations ^c^

^a^ Families referenced the fetus an average of 7 times total per consultation. ^b^ Clinicians referenced the fetus an average of 82 times total per consultation. ^c^ These instances were not mutually exclusive. Agency and/or mental states were used in 11 consultations total.

## Data Availability

De-identified data supporting the study findings are available upon request from the corresponding author. Data use agreements will need to be approved to access the data. The data are not publicly available due to privacy and ethical restrictions.
